# Histopathology Feature Mining and Association with Hyperspectral Imaging for the Detection of Squamous Neoplasia

**DOI:** 10.1038/s41598-019-54139-5

**Published:** 2019-11-28

**Authors:** Guolan Lu, Dongsheng Wang, Xulei Qin, Susan Muller, James V. Little, Xu Wang, Amy Y. Chen, Georgia Chen, Baowei Fei

**Affiliations:** 1 Department of Biomedical Engineering, Emory University and Georgia Institute of Technology, Atlanta, GA USA; 2 Department of Hematology and Medical Oncology, Emory University, Atlanta, GA USA; 3 Department of Radiology and Imaging Sciences, Emory University, Atlanta, GA USA; 4 Department of Otolaryngology, Emory University School of Medicine, Atlanta, GA USA; 5 Department of Pathology and Laboratory Medicine, Emory University School of Medicine, Atlanta, GA USA; 6 Department of Bioengineering, The University of Texas at Dallas, Richardson, TX USA; 7 Department of Radiology, The University of Texas Southwestern Medical Center, Dallas, TX USA

**Keywords:** Optical imaging, Oral cancer detection

## Abstract

Hyperspectral imaging (HSI) is a noninvasive optical modality that holds promise for early detection of tongue lesions. Spectral signatures generated by HSI contain important diagnostic information that can be used to predict the disease status of the examined biological tissue. However, the underlying pathophysiology for the spectral difference between normal and neoplastic tissue is not well understood. Here, we propose to leverage digital pathology and predictive modeling to select the most discriminative features from digitized histological images to differentiate tongue neoplasia from normal tissue, and then correlate these discriminative pathological features with corresponding spectral signatures of the neoplasia. We demonstrated the association between the histological features quantifying the architectural features of neoplasia on a microscopic scale, with the spectral signature of the corresponding tissue measured by HSI on a macroscopic level. This study may provide insight into the pathophysiology underlying the hyperspectral dataset.

## Introduction

More than half a million patients are diagnosed worldwide with squamous cell carcinoma (SCC) of the head and neck each year^1^. Only half of the people diagnosed with oral SCC live for five years^2^. The gold standard for cancer diagnosis remains tissue biopsy with pathological assessment made by pathologists using visual examination of haematoxylin and eosin (H&E) stained sections under the microscope^3^. However, the effectiveness of cancer diagnosis is highly dependent on the attention and experience of the pathologists. This technique is considered invasive, expensive, and time-consuming. The diagnosis and grading of oral epithelial dysplasia is based on a combination of architectural and cytological changes^3^, but evaluation of these changes is subjective and known to be inconsistent due to considerable inter- and intra-observer variations in the grading of lesions^4^. Digital pathology, which leverages the power of whole slide imaging and computer-aided diagnosis, holds great promise to providing rapid, consistent, and quantitative cancer diagnosis from histopathology images. Furthermore, noninvasive alternatives, such as various kinds of optical imaging techniques, have been sought to avoid the pain and discomfort of the biopsy procedures. Recent advancements in hyperspectral cameras, image analysis methods, and computational power have led to the development of hyperspectral imaging (HSI) system as a promising diagnostic tool for early cancer detection^5^.

HSI is an emerging optical modality that combines spectroscopy and wide-field imaging, which can rapidly interrogate large tissue surfaces and do not require tissue removal. Although spectroscopy has been explored for probing molecular, cellular, and tissue properties and characterizing correlation of tissue parameters with disease state, such fundamental research has not been investigated vigorously in HSI. Many existing studies have been focused on developing new hardware systems^5^. Very few efforts have been dedicated to investigate the underlying biological rationale of HSI for cancer detection, and the biological origins of differences in the measured reflectance signals of normal and neoplastic tissue are not well understood.

HSI provides an indirect measurement of the underlying tissue biochemical and morphological properties. The spectral characteristics of diffuse reflectance from heterogeneous biological tissue are the result of a complex interplay of the intrinsic absorption and scattering properties of the tissue^6,7^, the distribution of chromophores and scatterers, together with the source-tissue-detector geometry^8^. Thus, the biochemical and/or structural characteristics of the biological tissue determine the intrinsic absorption and scattering properties of the tissues, which in turn generate the measured reflectance signature. In the field of diffuse reflectance spectroscopy, researchers have reported a variety of modeling methods^9–13^ to inversely estimate the absorption and scattering coefficients *μ*_*a*_ and *μ*_*s*_, respectively, from the diffuse reflectance to characterize tissue properties. Palmer *et al*.^10^ developed a fast Monte Carlo-based inverse model of diffuse reflectance to extract the concentration of absorbers and the size and density of scatters present in human breast tissue samples, however, this model is limited in that it requires a priori knowledge of the absorbers and scatterers present in the tissue of interest. These modeling methods provide a way to connect the spectral features with the underlying biochemistry and morphology. However, they generally rely on the assumption of simplified tissue composition and structure, and specific source-detector settings.

To meaningfully interpret the hyperspectral dataset, there is a requirement to relate tissue architecture and morphology that occur with neoplasia to the bulk optical signal measured. On a microscopic scale, the architectural and cytological changes in neoplastic tissue can be quantified by a variety of histological features extracted from digitized pathological images for computer-aided diagnosis. Meanwhile, the spectral signature at each image point reflects the macroscopic features of corresponding tissue. Thus, we hypothesize that the spectral signature measured by HSI has significant association with histological features which quantify the tissue architectural and morphological alterations during neoplastic transformation. To validate this hypothesis, a predictive model is developed to combine multiple pathological features, including color, texture, morphometry, and topology features from epithelium tissue and its constituent nuclei and cytoplasm, for computer-aided diagnosis of tongue neoplasia. Next, an optimal feature subset is selected from these original histological features to best distinguish normal tissue from neoplastic tissue in histological images. The predictive performance of the optimal feature set is validated in histological images of both mouse tongue and human tongue. Finally, the correlation coefficients between the spectral signature of both *in vivo* and fresh *ex vivo* mouse tongues and the optimal histological features of the corresponding histological images are calculated and interpreted.

## Results

### Image analysis pipeline

**Figure 1:**
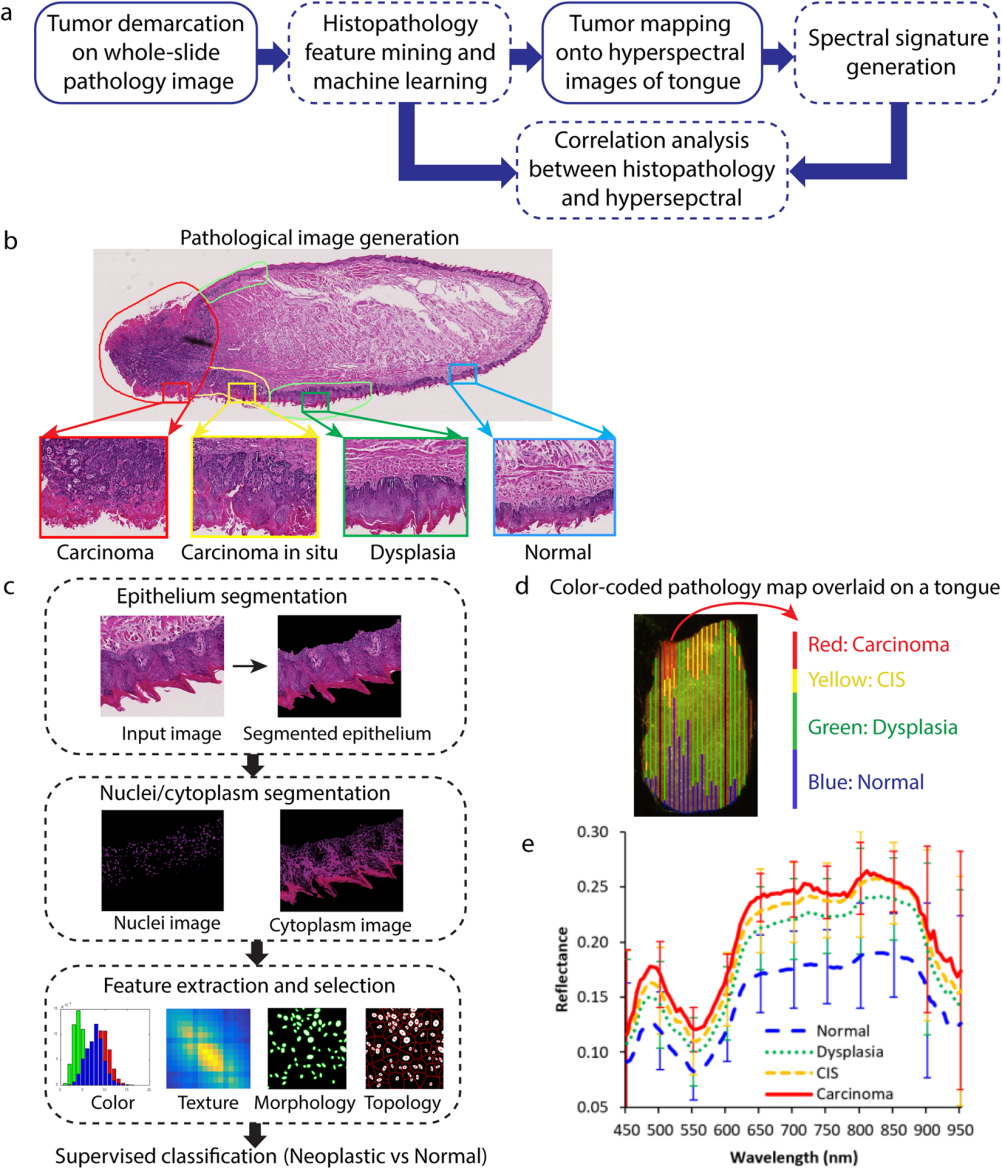
Method overview of the correlation analysis between spectral signature and histological features. (**a**) Summary of the quantitative image analysis pipeline. (**b**) An example of a whole-slide digital image of a tongue slice segmented into carcinoma, carcinoma *in situ*, dysplasia, and normal regions by an experienced pathologist. (**c**) Flowchart for pathological feature mining. (**d**) An example of the reconstructed pathology map color-coded and overlaid on a mouse tongue. (**f**) Reflectance spectral signature from hyperspectral images of a mouse tongue *in vivo*.

To identify the correlation between pathological features of tongue neoplasia and the corresponding spectral signatures, we developed an automatic image analysis pipeline (Fig. 1). The image analysis pipeline was validated the pipeline on pathological dataset from both mouse tongues and human tongues. Tissue slides of mouse tongues were obtained from a chemically-induced tongue carcinogenesis animal experiment, and corresponding hyperspectral images of the mouse tongue both *in vivo* and *ex vivo* were acquired with a hyperspectral imager as described in a previously published article^14^. Tissue slides of human tongues were obtained from surgical specimens of patients with tongue cancer in a clinical study^15^. Images of size 1472 × 922 were cropped from the whole-slide image at 20 × magnification using the Aperio ImageScope software (Leica Biosystems). A total of 1157 pathology images from 10 mouse tongue were generated (Table 1) and a total of 60 pathology images from 6 patients were generated (Table 2).

**Table 1:** Summary of Pathological Images from a Mouse Tongue Carcinogenesis Model.

Mouse ID	Normal	Dysplasia	CIS	Carcinoma	Total Number of Images
M1	45	68	25	20	158
M2	79	56	51	4	190
M3	49	51	21	0	121
M4	18	40	9	0	67
M5	80	92	10	0	182
M6	26	38	8	0	72
M7	29	56	0	0	85
M8	70	53	0	0	123
M9	13	52	0	0	65
M10	13	81	0	0	94
Total Number of Images	422	587	124	24	1157

**Table 2:** Summary of Pathological Images of Tongues from Human Patients.

Patient ID	Normal	CIS	Cancer	Total Number of Images
P1	3	8	4	15
P2	2	0	10	12
P3	1	0	4	5
P4	4	0	4	8
P5	9	0	3	12
P6	3	0	5	8
Total Number of Images	22	8	30	60

The first step of the pipeline involved demarcating tumor regions on whole-slide pathology images of tongue lesions (Fig. 1a). To do this, an experienced pathologist specialized in head and neck cancer outlined the regions of normal, dysplasia, carcinoma *in situ*, and carcinoma on the epithelium of the tongue on whole-slide digital images of H&E stained tissue slides (Fig. 1b). The second step was to identify the most discriminative pathological features for differentiating neoplasia from non-neoplastic tongue tissue through pathological feature mining and machine learning (Fig. 1c). This was done by first segmenting epithelium from the histological RGB image. In clinical practices, the diagnosis of precursor lesions is based on the altered epithelium with an increased likelihood for progression to squamous cell carcinoma^16^. Next, the generated epithelium image was further segmented into nuclei, cytoplasm and background. Then, a large number of image features were extracted from different histological components of each pathological tissue type, and an optimal feature subset was selected based on supervised learning to best distinguish between neoplastic and non-neoplastic tissue. The third step was to reconstruct the pathological tumor map for hyperspectral images of the tongue (Fig. 1d). Since each mouse tongue specimen were sectioned into a series of 5 µm slices with 200 µm interval (Fig. 1d), each H&E slide of the tongue (as shown in Fig. 1b) corresponded to a straight line on the dorsal surface. Regions of distinct pathology on each whole-slide image were mapped onto its corresponding straight line on top of the dorsal surface of the tongue to generate a color-coded pathology map for the corresponding tongue hypercube. As for human tongue, pathology tissue slice was in the same orientation as the specimen and affine registration was performed to directly map tumor region onto the patient tongue. Next, the reflectance spectra of individual pixels within each pathological category along each line were averaged to generate the spectral signature of normal, dysplasia, carcinoma *in situ*, and carcinoma tissue. Figure 1e showed representative spectral signatures of normal and neoplastic tissue (dysplasia/CIS/carcinoma) measured by HSI from mouse tongues *in vivo*. The overall shapes of these spectra appeared similar for all the pathology types, which were generally smooth and broad in the visible and near-infrared region. The characteristic dips around 540 nm coincided with hemoglobin’s absorption peaks. The reflectance intensity was generally weaker for healthy tissues than for neoplastic tissue including dysplasia, CIS, and carcinoma in both *in vivo* and *ex vivo* mouse tongues. Finally, the most discriminative pathological features were correlated with the spectral signatures from the same tongue to decipher the potential meaning of the spectral signatures. More details about the image analysis procedures were described in Methods section.

### Feature selection and predictive modeling

Multiple features were extracted from both the entire epithelial image and its constituent components including nuclei and cytoplasm. To quantify these abnormal tissue changes during carcinogenesis, 71 color and 149 texture features are extracted from each epithelium image and each cytoplasm image, respectively. To quantify these changes, 71 color, 149 texture, 44 morphometric, and 8 topological features were extracted from each nuclei image. The color and texture features were the same as extracted from the epithelium and cytoplasm image. In total, we extracted 712 features from each epithelium image and its constituent nuclei and cytoplasm images. A detailed summary of these features can be found in Supplementary Table 1.

Next, feature selection and supervised classification with nested cross validation was conducted to build predictive models for cancer diagnosis. The image set used for normal/neoplastic classification consisted of a total of 1157 images (735 neoplastic and 422 normal images) from 10 mouse tongues. To identify a compact and distinctive feature subset from the 721 features extracted from each histology image, we performed feature selection and built predictive models through nested cross validation (CV) consisting of leave-one-out outer CV and leave-one-out inner CV. The outer CV loop was used to estimate the classification performance; and the inner CV loop was used to tune the optimal parameters for the model development. Each run of the ten-fold outer CV algorithm consisted of training models on image set from nine tongues and testing on image set from the remaining tongue. A nine-fold inner CV was conducted to select the optimal feature numbers from the subsets of the training data from eight tongues and to validate the model using the remaining subset. Support vector machine with Gaussain Radial Basis (RBF) function was used as the classifier. Parameters were optimized via grid search over a pre-defined range. We considered the feature dimension *m* over the range of [1, 5, 10, 30, 50, 70, 90, 100, 200, 300, 342], and the cost values *c* ∈ 2^[−5, −3,−1, 1, 3, 5]^ and kernel parameters *γ* ∈ 2^[−5, −3, −1, 1, 3, 5]^. The model performance was evaluated with accuracy, sensitivity, and specificity.

**Figure 2:**
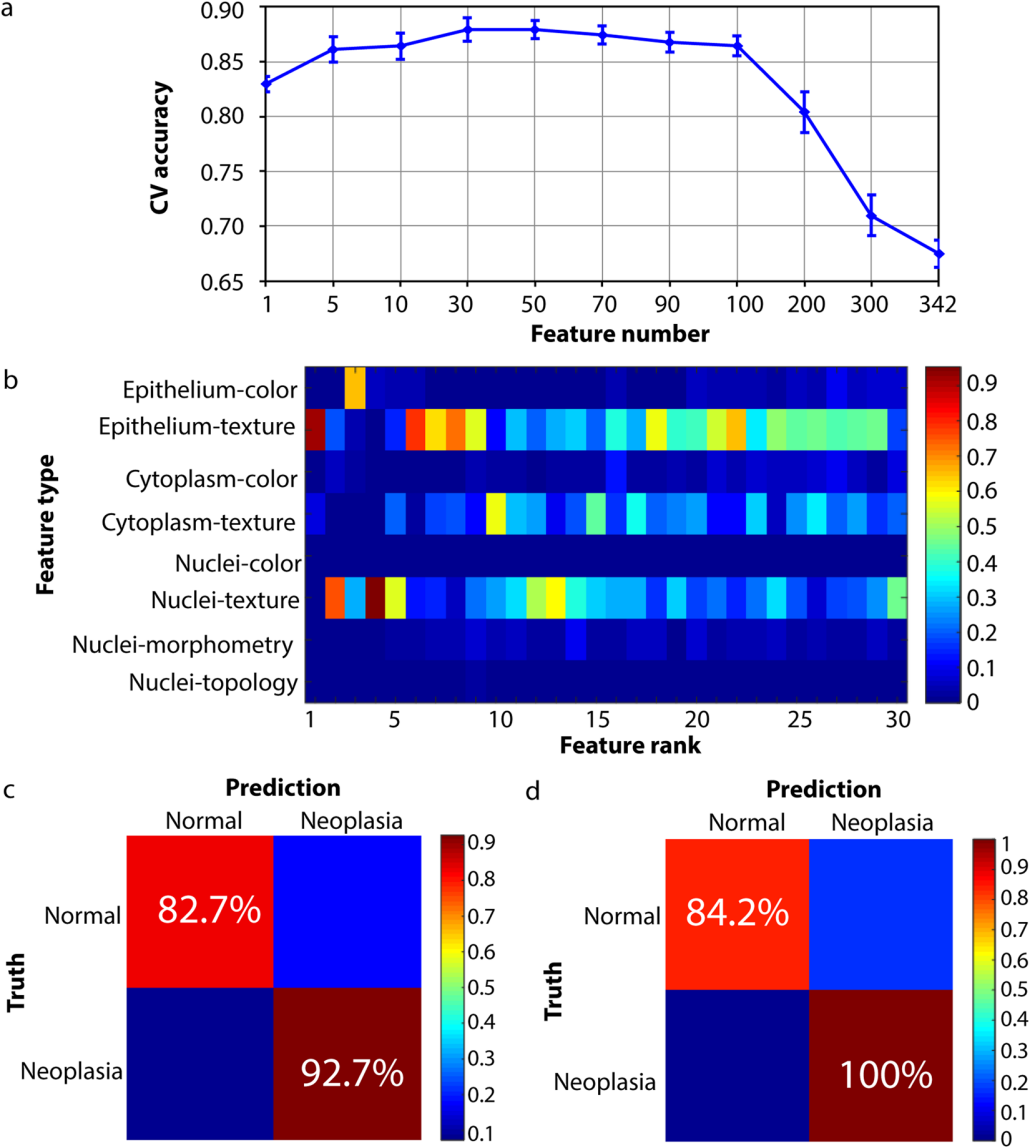
Feature selection and predictive modeling for the distinction of tongue neoplasia from non-neoplastic tissue. (**a**) The average CV accuracy of all samples as a function of feature dimensions. (**b**) Feature ranking frequency as a color heatmap. (**c**) Confusion matrix of prediction on mouse tongue pathology dataset. (**d**) Confusion matrix of prediction on human tongue pathology dataset.

Figure 2a plotted the mean CV accuracy of all samples as a function of feature dimension. We can see that when the number of features reached 30, classification accuracy reached the maximum. Since mRMR method worked by incrementally adding features according to maximal relevance and minimal redundancy criterion, we can further look at which feature was the most frequently selected to be the first, second, up until the 30^th^ feature during the cross validation. In Fig. 2b, the horizontal axis was the feature rank, and the vertical axis was feature categories. Bright color means the feature was highly selected. Among the best 30 features, texture features extracted from epithelium and its constituent components: cytoplasm and nuclei were the most frequently selected feature type across all the 30 ranks for the distinction of normal tissue from neoplasia. Mathematically, texture features characterize differences in the spatial arrangement of gray values of neighboring pixels, which have been shown to be effective at quantifying the tissue structural changes for oral cancer grading in [193]. Figure 2c showed the confusion matrix for neoplasia detection, which had a sensitivity of 92.7% and a specificity of 82.7%. To further validate the method performance in human patients, we extracted the best 30 features found in mouse tongue from pathological images of six human tongue cancer patients, and ran leave-one-out cross validation. Figure 2d displayed the confusion matrix for human tongue dataset with a sensitivity of 100% and specificity of 84.2%. This demonstrated that the discriminative pathology features extracted from mouse tongue is also highly discriminative in classifying human tongue lesions.

### Correlation between spectral signature and pathological features

**Figure 3:**
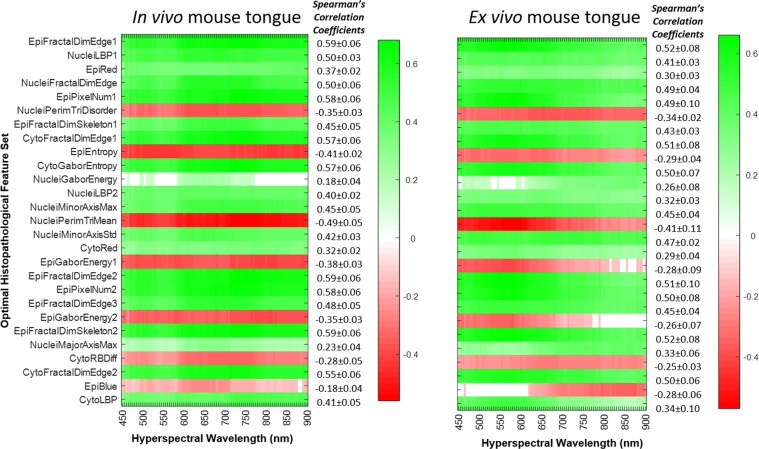
Correlation heatmap showing Spearman’s correlation coefficient between spectral signature (horizontal axis) and the selected optimal histology feature subset (vertical). Green = positive correlation, red = negative correlation, white = no correlation or correlations that are not statistically significant.

Figure 3 illustrated the correlation heatmap of pairwise association between the optimal histological feature subsets which were found to have the best distinguishing power for neoplasia detection and spectral signature from hyperspectral imagers of mouse tongues *in vivo and ex vivo*. The average and standard deviation of the correlation coefficients for each row were listed next to the corresponding heatmap. We had three major observations from the heatmaps. Firstly, spectral signature from HSI was significantly associated with the optimal histological feature set, which suggested that HSI captures the key diagnostic information reflecting the tissue architectural and morphological changes during neoplastic transformation. Secondly, the strengths of the correlation between each histology feature and reflectance intensities over all the wavelengths were very similar, which suggested that the diagnostic importance of the whole spectral information for cancer detection. Lastly, the spectral signature of *in vivo* tongues exhibited stronger association with histological features than that of *ex vivo* tongues, which suggests that hyperspectral images of *in vivo* tongues capture more diagnostic information than those of *ex vivo* tongues.

Table 3 summarized all the nine histology features which were highly and significantly associated with spectral signature (average correlation coefficients *r*_*s*_ ≥ 0.5 or *r*_*s*_ ≤ −0.5). Seven out of the nine features are quantifying the textural changes in tissue. Fractal dimension extracted from the epithelium, nuclei, and cytoplasm were the most frequently selected features with strong and significant correlation with spectral signature. Only one histology feature had strong enough negative correlation: the mean perimeter of the Delaunay Triangulation constructed from the segmented nuclei image.

**Table 3:** Summary of Representative Histological Features ($\Vert {{r}}_{{s}}\Vert \ge 0.5$).

Location	Feature Name	*r*_*s*_	Feature Explanation
Epithelium	Fractal dimension (edge)	0.59	Quantitative description of complex, irregularly shaped objects in epithelium
	Fractal dimension (skeleton)	0.59	Quantitative description of complex, irregularly shaped objects in epithelium
	Pixel number (edge)	0.58	Quantitative description of complex, irregularly shaped objects in epithelium
Nuclei	Fractal dimension (edge)	0.59	Quantitative description of complex, irregularly shaped nuclear objects
	Local binary pattern	0.5	Rotation-invariant texture feature characterizing spatial structure and contrast of nuclei
	Minor axis length (max)	0.45	Quantify the variations in nuclear size and shape
Cytoplasm	Fractal dimension (edge)	0.57	Quantitative description of complex, irregularly shapes in cytoplasm
	Gabor texture (entropy)	0.57	Characterize the randomness in texture of Gabor magnitude of cytoplasm image
Nuclei	Perimeter of Delaunay triangulation (mean)	−0.49	Describe the distances between individual nuclei

**Figure 4:**
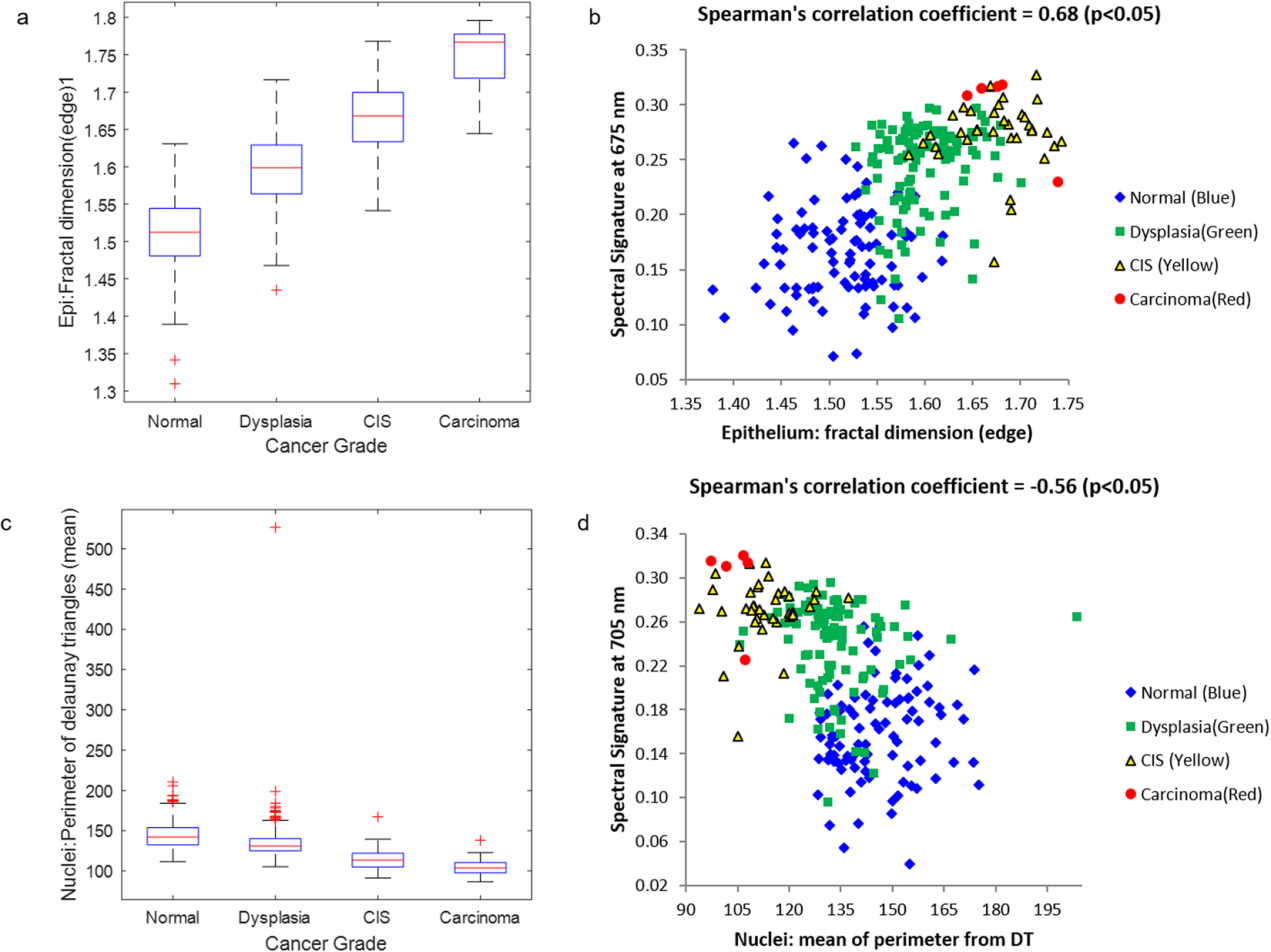
Distribution of histological features and corresponding scatter plots with spectral signature at selected wavelengths. In (**a**,**b**), the histology feature is fractal dimension extracted from epithelium, which has strong and significant correlation coefficients with spectral signature at 715 nm. In (**c**,**d)**, the histology feature is the mean perimeter of Delaunay triangles extracted from nuclei image, which exhibits significant negative correlation with spectral signature at 745 nm.

Furthermore, we plotted the distribution of two representative histology features with significant and strong positive and negative correlation with spectral signatures as shown in Fig. 4a,c. As the degree of tissue malignancy increases, the fractal dimension of the epithelium tends to increase (Fig. 4a), which reflects the abnormal structural and morphological changes of tissue during neoplastic transformation, such as the loss of cellular organization, increase atypical nuclei^3^, etc. In the meanwhile, as the fractal dimension of the epithelium increases, the reflectance intensities also tends to increase as shown in Fig. 4b, which shows positive correlation between histology feature and spectral signature. On the other hand, the mean perimeter of the Delaunay triangles tends to decrease as tissue transform from benign to malignancy (Fig. 4c), which is consistent with the fact that nuclei have proliferated and become more crowded in malignant tissue. As shown in Fig. 4d, the reflectance intensity tends to increase while the mean perimeter of Delaunay triangles decreases, exhibiting negative association between the two.

## Discussion

The major contribution of this study was the successful validation of the hypothesis that the spectral signature has significant association with histologic features that reflect the tissue architectural changes during malignant transformation. Multiple quantitative histologic features were extracted and selected from the epithelium and its constituent components-nuclei and cytoplasm, which best distinguished normal from neoplastic tissue for both mouse tongue and human tongue. The selected optimal feature subset was a combination of color, texture, morphometry, and topology features, which quantified the architectural and morphological changes during tissue malignant transformation. We noted that the average reflectance intensities of neoplasia were stronger than that of normal tissue in both *in vivo* and *ex vivo* mouse tongues. This observation is consistent with the representative *in vivo* reflectance spectra of nonmalignant and malignant tissue from patients with head and neck cancers^17^, and is also consistent with the *ex vivo* reflectance spectra of malignant and adjoining normal tissue from the tongue cancer patients^18^.

Furthermore, we also observed for the first time that reflectance intensity would increase with increased fractal dimension and other texture features throughout neoplastic progression. The increase of fractal dimension is associated with the structural changes in epithelium, such as increased nuclear size, atypical nuclear shape, increased DNA content, and hyperchromasia with coarse and irregular chromatin clumping. So one possible explanation for the spectral difference between normal and neoplastic tongue tissue could be that light scattering events inside the epithelium tissues change significantly with the progressive development of squamous lesions, thus leading to the alterations in the diffuse reflectance spectrum and forming a potential physiologic basis for cancer detection with hyperspectral imaging. More specifically, the scattering coefficient *μ*_*s*_ (in units of mm^−1^) is a quantitative measure of radiant energy loss caused by tissue scattering^8^. Qualitatively, for a given tissue volume and fixed absorption conditions, the diffuse reflectance increases as tissue scattering *μ*_*s*_ increases^8^. Therefore, the alteration of the scattering density and distribution in the epithelium is likely to be contributing to the increased reflectance in neoplastic tissue. The scattering is relatively homogeneous across all the wavelengths, which may explain the homogeneous association between spectral signature and individual histological features.

**Figure 5:**
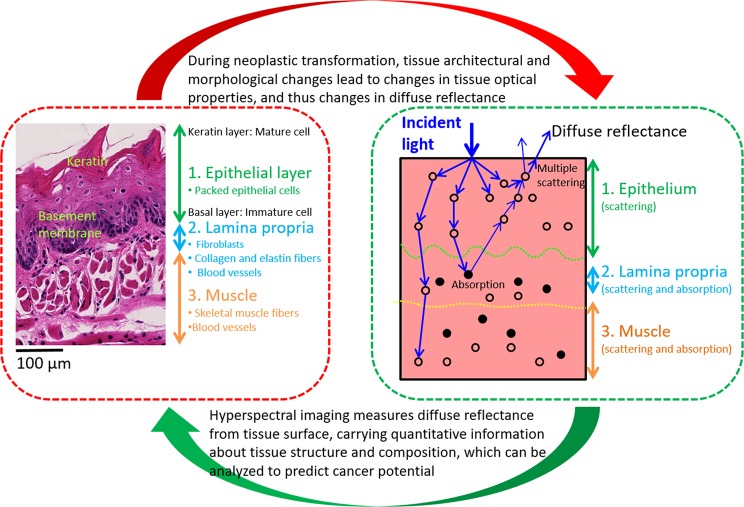
Interpretation of the association between HSI and histological features.

To better understand the scattering origin in tongue, we further looked into the histological structures of the tongue (Fig. 5). Our study shows that mouse tongues have layered structures similar to human tongues, which consists of an outer stratified squamous epithelial tissue that is linked to a dense connective tissue (i.e. lamina propria) and deep skeletal muscle fibers. According to^19^, the epithelium generally consists of several cellular layers of epithelial cells: it starts from the innermost basal layers of undifferentiated cells, then moves to suprabasal cell layers, and ends at the outermost keratinized layers of the mature cells. The tissue components in lamina propria mainly include fibroblasts, collagen fibers, and blood vessels (capillaries). The skeletal muscle mainly includes bundles of striated muscles with many blood vessels and nerves between them.

In the therapeutic window from about 600 to 1300 nm, biological tissue have relatively low absorption and scattering, thus allowing maximum depth of penetration into biological tissue and high reflectance out of tissue^8^. Biological tissue is highly scattering, because cell membranes and various organelles have different refractive indices compared to the cytoplasm, and the extracellular matrix is also highly scattering. The total tissue reflectance can be from two sources: one is the single backscattering mainly from the epithelium and the other is the multiple scattering events arising from the deeper tissue structures. As tissue transform from benign to malignant tumors, a series of cytological and architectural changes occur progressively, which in turn alters the distribution, density, size, and shape of major light scatters such as collagen, keratin, cell nuclei, mitochondria, and other cytoplasmic organelles during cancer progression. All these architectural and cytological changes could affect the tissue scattering properties and thus change the reflectance measured by HSI. In the dysplastic tissue, the loss of cell maturation from the basement membrane to the surface is a major morphological change^20^. This study suggested that the structural changes of the neoplastic tongue such as increased epithelium thickness and increased nuclear density may increase the backscattering of light, and the increased scattering may lead to the spectral difference between neoplastic and healthy tissue. These hypotheses remain to be investigated by future studies.

In summary, we have demonstrated that the spectral difference between normal and neoplastic tissue of mouse tongues, as measured by HSI in a macroscopic scale, is associated with the histological features that can quantify the tissue architectural alterations during neoplastic transformation. This study provided some insights into the interpretation of cancer data acquired with hyperspectral imaging.

## Methods

### Hyperspectral image acquisition

#### Instrumentation

A CRI Maestro hyperspectral imaging camera system (PerkinElmer Inc., Waltham, Massachusetts) was used to collect reflectance images from 450 nm to 900 nm with 5 nm increments in this study. This instrument is comprised of a flexible fiber-optical light system with a Xenon light source, a solid-state liquid crystal tunable filter (LCTF) as the wavelength dispersion device, a spectrally optimized lens, and a 12-bit charge-coupled device (CCD) as the area detector.

#### Animal data collection

Animal data was collected using our protocol that was previously described^14^. Briefly, female CBA/J mice purchased from Jackson Laboratory (Bar Harbor, Marine) were treated with drinking water mixed with 4-nitroquinoline-1-oxide (4NQO) powder (Sigma Aldrich, Saint Louis, Missouri) for 16 consecutive weeks (concentration: 100 μg/mL) in order to induce tongue carcinogenesis. These mice were monitored weekly for body weight and water consumption, and the experiment was terminated at 24 weeks. All of the animal procedures were conducted in accordance with the Guidelines for the Care and Use of Laboratory Animals and were approved by the Institutional Animal Care and Use Committee (IACUC) of Emory University. All methods were performed in accordance with relevant guidelines and regulations.

Before imaging these mice, we first acquired white and dark reference hypercubes. White reference image cubes are acquired by placing a standard white reference board in the field of view. The dark reference cubes are acquired by keeping the camera shutter closed in absence of light. Next, we anesthetized the mice with ketamine and acquired hyperspectral images of the mouse tongues *in vivo* in a supine position. Then, we euthanized the mouse by cervical dislocation and procured the tongue specimen for *ex vivo* hyperspectral imaging. Immediately after *ex vivo* imaging, each dissected tongue was formalin fixed and paraffin embedded and then cut into a series of 5 μm tissue slices across the entire tongue. These tissue slides were H&E stained and digitized for pathology diagnosis. A clinically experienced pathologist (SM) reviewed the H&E slides and graded the dorsal surface of each tongue slice as regions of normal (including healthy and hyperplastic tissue), dysplasia, CIS, and SCC^3^. Finally, we reconstructed the pathology map of the tongue by mapping the histology slides of the tongue cross-sections back to the tongue surface. In this way, the tissue position where the spectrum was captured can be matched with specific pathological diagnosis.

#### Human sample collection

Human samples were collected using our clinical protocol that was previously described^15^. Briefly, surgical specimens were collected from patients (n = 6) with head and neck cancers who were consented for our study under the Head and Neck Satellite Tissue Bank (HNSB, IRB00003208) protocol approved by the Emory University Institutional Review Board (IRB). The human subjects of the surgical specimens provided the written, informed consent before the study. The samples were de-identified before being released to our laboratory. During the surgery, resected specimens were sent to the pathology room for margin assessment. Three tissue samples including clinically visible tumor, clinically normal tissue, and tumor-normal interface (tumor with adjacent normal tissue), were procured from the main specimen of each consented patient. Specimen collection and imaging did not affect the procedure time in the operating room (OR) or the content and verification of the final pathology report.

Before imaging the tissue sample, white and dark reference hypercubes were acquired as described above. Next, the specimens were placed on a non-reflective blackboard on the imaging stage. Reflectance hyperspectral images of the specimen from the side-view (not vertically from epithelium to deeper tissue as in mouse tongue) were obtained from 450–900 nm with 5-nm intervals. After all the imaging procedures were finished, tissue specimen were formalin-fixed overnight and then sent to the Pathology Department for standard histologic processing. Two to three serial sections with 5 µm thickness from the imaging surface were cut and H&E. These tissue sections were then digitally scanned for the pathology diagnosis. A clinically experienced pathologist examined the histology slides and outlined the tumor boundary on the digitized slides as the gold standard. All methods were performed in accordance with relevant guidelines and regulations.

#### Hyperspectral image pre-processing

The acquired hyperspectral data was converted into relative reflectance by a white reference image and a dark reference image with the following equation:

$${I}_{ref}(x,y,\lambda )=\frac{{I}_{raw}(x,y,\lambda )-{I}_{dark}(x,y,\lambda )}{{I}_{white}(x,y,\lambda )-{I}_{dark}(x,y,\lambda )},$$

where *I*_*ref*_(*x*, *y*, *λ*) is the normalized reflectance value at the pixel location (*x*, *y*) and the wavelength band *λ*. (*x*, *y*, *λ*) is the raw intensity value of a sample pixel (x, y). *I*_*wh*_(*x*, *y*, *λ*) and *I*_*dark*_(*x*, *y*, *λ*) are the corresponding pixels from the white and dark reference images at the same wavelength as the sample image.

### Histological image segmentation

#### Segmentation of epithelium

To pre-process histological images for feature extraction, we first segment the epithelium tissue from the connective tissue, muscle, and background (see Supplementary Fig. 1). The grayscale image extracted from the red color channel exhibits the best contrast for separating epithelial layer from the underlying connective tissue, as compared with the luminescence image of the HSV color space, and green and blue channel images from the RGB color space. Therefore, to segment out the epithelial layer, the red channel image is smoothed with edge-preserving filters^21^ and then binarized with global thresholding. Finally, morphological post-processing was conducted to fill the holes in the epithelium and remove small spurious background regions with morphological opening.

#### Segmentation of nuclei and cytoplasm

H&E staining of a tongue tumor histological image enhances three colors: blue-purple, pink, and white. These colors correspond to specific cellular structures. Basophilic structures containing nucleic acids-ribosome and nuclei-tend to stain blue-purple; eosinophilic intra- and extracellular proteins in cytoplasmic regions tend to stain bright pink; empty spaces do not stain and tend to be white. The colors consisting of blue-purple, pink, and white in the histological image allows a clear distinguishing between different cellular components within the epithelium. Therefore, k-means clustering using the Euclidean distance is applied on RGB color image of the epithelial layer to segment the epithelium into its three constituent components: nuclei, cytoplasm, and background.

The initial nuclear masks generated by k-means clustering suffer from the problems of small spurious background noise, small holes within the nuclei region due to nuclear inhomogeneity, and nuclear clusters with overlapping nuclei. To address the first two problems, a series of morphological operations are conducted: First, the holes on nuclei mask are filled; Second, the filled nuclear mask is dilated with a disk radius of 2. Third, morphological opening with a disk radius of 4 is applied and connected components with fewer than 50 pixels are discarded. After the morphological processing, pure nuclei images are generated, but some nuclei are still overlapping, including large and small nuclear clusters. To obtain the mask for individual nuclei, our strategy is to first break down large nuclear clusters into smaller nuclear clusters, and then segment smaller nuclear clusters into individual nuclei.

To break down the large nuclear clusters, we first conduct repeated k-means clustering with 3 steps: The first step is to identify the large nuclear clusters with solidity lower than 0.9, and with more than 500 pixels. The second step is to apply k-means clustering on the large nuclear clusters to generate individual nuclei and small nuclear clusters, with fewer large nuclear clusters left. The third step is to repeat the first two steps until all the individual nuclei were segmented. Since nuclei color is dominated by blue, any nucleus with red to blue ratio larger than 1.2 is considered as false nucleus detection and thus is removed from the nuclear image. Finally, morphological operation is applied to fill the holes within the nuclear mask, and areas with less than 25 pixels were discarded.

To further segment the small nuclear clusters into individual nuclei, the following five-step procedure is repeated twice: The first step is to identify small nuclear clusters more than 150 pixels and lower than 0.9 solidity. The second step is to convert the RGB image of these small nuclear clusters into a blue ratio image with the following equation: $\frac{Blue/(1+Rlue+Green)}{(1+Red+Green+Blue)}$. The third step is to smooth the blue ratio image with edge-preserving filtering. The fourth step is to run marker-controlled watershed segmentation on the smoothed blue ratio image to separate the touching nuclei. The final step is to fill the holes within the nuclear mask, and areas with less than 50 pixels were discarded.

After all the touching nuclei are segmented, the single nuclear masks from all previous steps are combined to form a mask for all the nuclei. Nuclear regions with average red to blue ratio higher than 1 are removed from the nuclear mask. Ellipse fitting is then conducted to get smooth contour of individual nuclei.

### Feature extraction

Multiple features were extracted from the epithelium, nuclei, and cytoplasm image to quantify the abnormal tissue changes during carcinogenesis as described below:

#### Color features

Visual examination of histological images reveals noticeable color changes during neoplastic transformation, which may be attributed to the invasion of cancer cells from epithelium layer where nuclei stains blue-purple into the stroma tissue which stains pink^22^. The pattern of color changes due to the proliferation and abnormal distribution of epithelial nuclei may be captured by the color channel histogram of the image. As a pre-processing step, we first transform the image from the RGB color space into the YCbCr space and then applying a threshold value of 180 to the luminance (Y) component. This is because it has been shown in previous study that removing white pixels could improve the classification performance^22^. Next, we extract the following color features:

Transformed RGB histogram: RGB histogram itself is sensitive to photometric variations. However, scale-invariance and shift-invariance with respect to light intensity can be achieved by normalizing the pixel value distribution as follows^23^:

$$(\begin{array}{c}R^{\prime} \\ G^{\prime} \\ B^{\prime} \end{array})=(\begin{array}{c}\frac{R-{\mu }_{R}}{{\sigma }_{R}}\\ \frac{G-{\mu }_{G}}{{\sigma }_{G}}\\ \frac{B-{\mu }_{B}}{{\sigma }_{B}}\end{array})$$

where *μ*_*X*_ is the mean and *σ*_*X*_ is the standard deviation of the distribution in color channel *X* (*X* = *R*, *G*, *B*) over the area under consideration. Transformed RGB histogram was obtained using 16 bins per color channel, yielding 48 features each indicating the proportion of pixels in the corresponding bin.

Red-Blue channel difference: we extracted a 16-bin histogram of the red-blue intensity difference, and 7 statistical measures (mean, median, standard deviation, minimum, maximum, skewness, kurtosis) of the red-blue intensity difference.

#### Texture features

Texture features characterize differences in the spatial arrangement of gray values of neighboring pixels. Here, we constructed a texture feature set including Gray Level Co-Ocurrence matrices (GLCM) feature, Gabor filters, Local binary pattern^24^, and Fractal textures^25^.

GLCM features: The gray-level co-occurrence matrix can reveal certain properties about the spatial distribution of the gray levels in the image^26^. For example, if the entries in the GLCM diagonal data are large, the regions are contiguous and the texture is coarse. With a small offset and the large concentrated entries, each diagonal element represents an image area of the corresponding gray-level region of interest. GLCM is an N-dimensional square matrix square, where N is the number of gray levels in the image. Element [*i*, *j*] of the matrix is generally by counting the number of times a pixel with value *i* is adjacent to a pixel with value *j* and then dividing the entire matrix by the total number of such comparisons made. Each entry is therefore considered to be the probability that a pixel with value *i* will be found adjacent to a pixel of value *j*.

To represent a grayscale image by GLCMs, we first scale the intensity values in the original image into 64 gray levels to reduce computational cost. Next, offsets need to be set to define pixel relationship of varying direction and distance. We generate 16 GLCMs with an array of offsets that specify four directions (horizontal, vertical, left and right diagonals), and four distances between pixels. Then we average all the GLCMs over each grayscale histology image and extract the 13 Haralick texture features^27^ and 6 other features^28^ (autocorrelation, cluster prominence, cluster shape, dissimilarity, inverse difference, and maximum probability) from the averaged GLCMs.

Gabor filter features: It is known that the human visual system processes visual information by decomposing the retinal image into a number of filtered images, each of which contains intensity variations over a narrow range of frequency (size) and orientation. Gabor filters utilize a multi-channel filtering approach to decompose the original image into several filtered images, and are therefore a useful model for texture discrimination^29^. A two-dimensional Gabor function is composed of a sinusoidal signal of some frequency and orientation, modulated by a Gaussian envelope. According to the parameter setting in^29^, we designed 28 Gabor filters by varying an orientation parameter $\theta =\{0,\frac{\pi }{4},\frac{\pi }{2},\frac{3\pi }{4}\}$ and a radial frequency parameter ${u}_{0}\in \{2\sqrt{2},4\sqrt{2},\,\ldots \,,128\sqrt{2}\}$. Next, we apply the Gabor filter array on each histological image, and extract gabor magnitude response from the filtered image. Finally, the entropy and energy of the gabor magnitude image are calculated as the Gabor texture features.

Local binary patterns (LBP): LBP is a robust and efficient texture descriptor for a wide range of applications in texture discrimination^30^. Here we use a unifosssrm rotation-invariant LBP with a neightborhood of 8 sampling points.

Fractal textures: Fractal geometry provides a tool for quantitative description of complex, irregularly shaped objects in pathological images^22^. A common fractal property of an object is its fractal dimension. The fractal dimension provides a quantitative measure of the space-filling capacity of an object. For instance, the fractal dimension of a straight line is the same as its topological dimension, i.e., 1, since it can only fill a one-dimensional subspace. In this study, we propose a modified segmentation-based fractal texture analysis (mSFTA) based on the SFTA method in^31^. The mSFTA is a two-step method. The first step is to decompose the input image into a set of binary images which the fractal dimension of the resulting regions are computed in order to describe segmented texture patterns. The set of threshold values is obtained by utilizing the multi-level Otsu algorithm^32^ to generate the first set of binary images until the desired number of thresholds *n*_*t*_ is obtained. Next, generate the second set of binary images by selecting *n*_*t*_ pairs of contiguous thresholds. After this step, the number of resulting binary images is 2*n*_*t*_. Here, *n*_*t*_ is set to be 4, so a total of 8 binary images were generated. This multi-threshold method allows the extraction of region information with different brightness levels. The second step is to extract fractal features that measure the complexity, size, and brightness of the 2*n*_*t*_ thresholded images. We first extract the boundary and skeleton of the binary images, and generate 2*n*_*t*_ binary images. Next we calculate the fractal dimension and the pixel size of all the binary images, as well as the mean intensity level and entropy of the corresponding grayscale boundary and skeleton image.

#### Morphometric features

Morphometric features are extracted from each segmented nuclei image, including the statistical measures (mean, median, std, min, max, skewness, kurtosis) of 7 size and shape features (area, major and minor axis length, nuclei solidity, eccentricity, compactness, and neighborhood radius), and the nucleus to cytoplasm ratio.

#### Topology features

The Delaunay graph is constructed based on the centroids of connected components segmented in the nuclei image^33^. We measure the statistics (mean, maximum, minimum, and disorder) of the area and perimeter of Delaunay triangles to characterize the distribution of individual nuclei.

### Feature selection

The goal of feature selection is to find a feature set S with n feature {λ_i_}, that “optimally” characterize the difference between cancerous and normal tissue. To achieve the “optimal” condition, we used the maximal relevance and minimal redundancy (mRMR)^34^ framework to maximize the dependency of each feature on the target class labels (tumor or normal), and minimize the redundancy among individual features simultaneously. Relevance is characterized by mutual information *I* (x; y), which measures the level of similarity between two random variables *x* and *y*:

1 $${\rm{I}}({\rm{x}};{\rm{y}})=\iint p(x,y)\log \,\frac{{\rm{p}}({\rm{x}},{\rm{y}})}{{\rm{p}}({\rm{x}}){\rm{p}}({\rm{y}})}{\rm{dxdy}}$$

where p(x, y) is the joint probability distribution function of *x* and *y*, and p(x) and p(y) are the marginal probability distribution functions of *x* and *y* respectively.

We represent the feature of each pixel with a vector ${\rm{\lambda }}=[{{\rm{\lambda }}}_{1},{{\rm{\lambda }}}_{2},\,\ldots \,,{{\rm{\lambda }}}_{{\rm{i}}}]$, i = 738 and the class label (tumor or normal) with c. Then the maximal relevance condition is:

2 $$\max \,{\rm{D}}({\rm{s}},{\rm{c}}),\,{\rm{D}}=\frac{1}{|{\rm{S}}|}{\sum }_{{{\rm{\lambda }}}_{{\rm{i}}}\in {\rm{S}}}{\rm{I}}({{\rm{\lambda }}}_{{\rm{i}}},{\rm{c}})$$

The feature set selected by maximal relevance is likely to have redundancy, so the minimal redundancy condition is used to select mutually exclusive features:

3 $$\max \,R(s),{\rm{R}}=\frac{1}{{|{\rm{S}}|}^{2}}{\sum }_{{{\rm{\lambda }}}_{{\rm{i}}},{{\rm{\lambda }}}_{{\rm{j}}}\in {\rm{S}}}{\rm{I}}({{\rm{\lambda }}}_{{\rm{i}}},{{\rm{\lambda }}}_{{\rm{j}}})$$

So the simple combination (Eq. (5) and (6)) of these two conditions forms the criterion “minimal-redundancy-maximal-relevance” (mRMR).

4 $$\max ({\rm{D}}-{\rm{R}})$$

i.e.

5 $$\max ({\sum }_{{{\rm{\lambda }}}_{{\rm{i}}}\in {\rm{S}}}{\rm{I}}({{\rm{\lambda }}}_{{\rm{i}}},{\rm{c}})-\frac{1}{|{\rm{S}}|}{\sum }_{{{\rm{\lambda }}}_{{\rm{i}}},{{\rm{\lambda }}}_{{\rm{j}}}\in {\rm{S}}}{\rm{I}}({{\rm{\lambda }}}_{{\rm{i}}},{{\rm{\lambda }}}_{{\rm{j}}}))$$

### Correlation analysis between spectral signature and histological features

Spearman’s rank correlation coefficient is a nonparametric approach for evaluating the degree of monotonic association or correlation between two independent variables. It assesses how well an arbitrary monotonic function can describe the relationship between two variables, without making any assumption about the frequency distribution of the variables. It is similar to Pearson’s product moment correlation coefficients except that it operates on the ranks of the data rather than the raw data. Spearman’s rank correlation has two major advantages over Pearson’s correlation. First, it has no assumption of normality for the dataset and is unaffected by the distribution of the population. Second, it is robust to outliers since it operates on the ranks of the data. Therefore, we use Spearman’s rank correlation coefficient to assess the association between spectral signatures and the selected optimal histology feature subset from all pathological tissue types. To test the significance of each pairwise correlation, we assume that there is no correlation between the two variables and reject the null hypothesis when p value is less than or equal to the significance level *α* = 0.05. The resulting matrix of pairwise correlation coefficients is visualized as a heatmap, where the positive and negative correlation coefficients are displayed in green and red, respectively. Correlation coefficients that are not statistically significant are displayed in white. All statistical analysis were performed in Matlab R2015b.

## Supplementary information

Supplementary material

## Data Availability

Images and data are anonymized and may be available for research upon request.
