# Supplementary material for: Histopathology Feature Mining and Association with Hyperspectral Imaging for the Detection of Squamous Neoplasia
Source: Sci Rep. 2019 Nov 28;9:17863. doi: 10.1038/s41598-019-54139-5 (PMC6882850; doi:10.1038/s41598-019-54139-5)
Supplement: Supplementary file 1 — Supplementary material [file 41598_2019_54139_MOESM1_ESM.docx]

**Histopathology Feature Mining and Association with Hyperspectral Imaging for the Detection of Squamous Neoplasia**

Guolan Lu^1^, Dongsheng Wang ^2^, Xulei Qin ^3^, Susan Muller ^4^, James V. Little^5^,

Xu Wang ^3^, Amy Y. Chen ^4^, Georgia Chen ^3^, and Baowei Fei ^1,3,6,7 *^

^1^ Department of Biomedical Engineering, Georgia Institute of Technology and Emory University, Atlanta, GA, USA

^2^ Department of Hematology and Medical Oncology, Emory University, Atlanta, GA, USA

^3^ Department of Radiology and Imaging Sciences, Emory University, Atlanta, GA, USA

^4^ Department of Otolaryngology, Emory University School of Medicine, Atlanta, GA, USA

^5^ Department of Pathology and Laboratory Medicine, Emory University School of Medicine, Atlanta, GA, USA

^6^ Department of Bioengineering, The University of Texas at Dallas, Richardson, TX, USA

^7^ Department of Radiology, The University of Texas Southwestern Medical Center, Dallas, TX, USA

*^*^ Corresponding author: Dr. Baowei Fei, E-mail:* [*bfei@utdallas.edu*](mailto:bfei@utdallas.edu)*, Website:* [*www.fei-lab.org/baowei-fei/*](http://www.fei-lab.org/baowei-fei/)

**Supplementary Materials**

**Table 1**. Summary of All the Histological Features

| **Tissue Components** | **Feature Name** | **Feature Type** | **Feature Number** |
| --- | --- | --- | --- |
| Epithelium | ***Color***: Transformed RGB histogram; Red-blue difference histogram; Statistical measures (mean, median, standard deviation, minimum, maximum, skewness, kurtosis) of the red-blue intensity difference. | color, texture | 220 |
|  | ***Texture***: Haralick feature, Local binary pattern, Fractal textures, Gabor filter (energy, entropy) |  |  |
| Cytoplasm | ***Color***: Transformed RGB histogram; Red-blue difference histogram; Statistical measures (mean, median, standard deviation, minimum, maximum, skewness, kurtosis) of the red-blue intensity difference. | color, texture | 220 |
|  | ***Texture***: Haralick feature, Local binary pattern, Fractal textures, Gabor filter (energy, entropy) |  |  |
| Nuclei | ***Color***: Transformed RGB histogram; Red-blue difference histogram; Statistical measures (mean, median, standard deviation, minimum, maximum, skewness, kurtosis) of the red-blue intensity difference. | color, texture, morphometry, topology | 272 |
|  | ***Texture***: Haralick feature; Local binary pattern; Fractal textures; Gabor filter. |  |  |
|  | ***Morphometry***: Statistical measures (mean, median, standard deviation, minimum, maximum, skewness, kurtosis) of the nuclear size, solidity, eccentricity, major axis length, minor axis length, compactness, neighborhood radius; Nuclei to cytoplasm ratio; |  |  |
|  | ***Topology***: Statistical measure (mean, maximum, minimum, disorder) of the area and perimeter of the Delaunay triangulation |  |  |


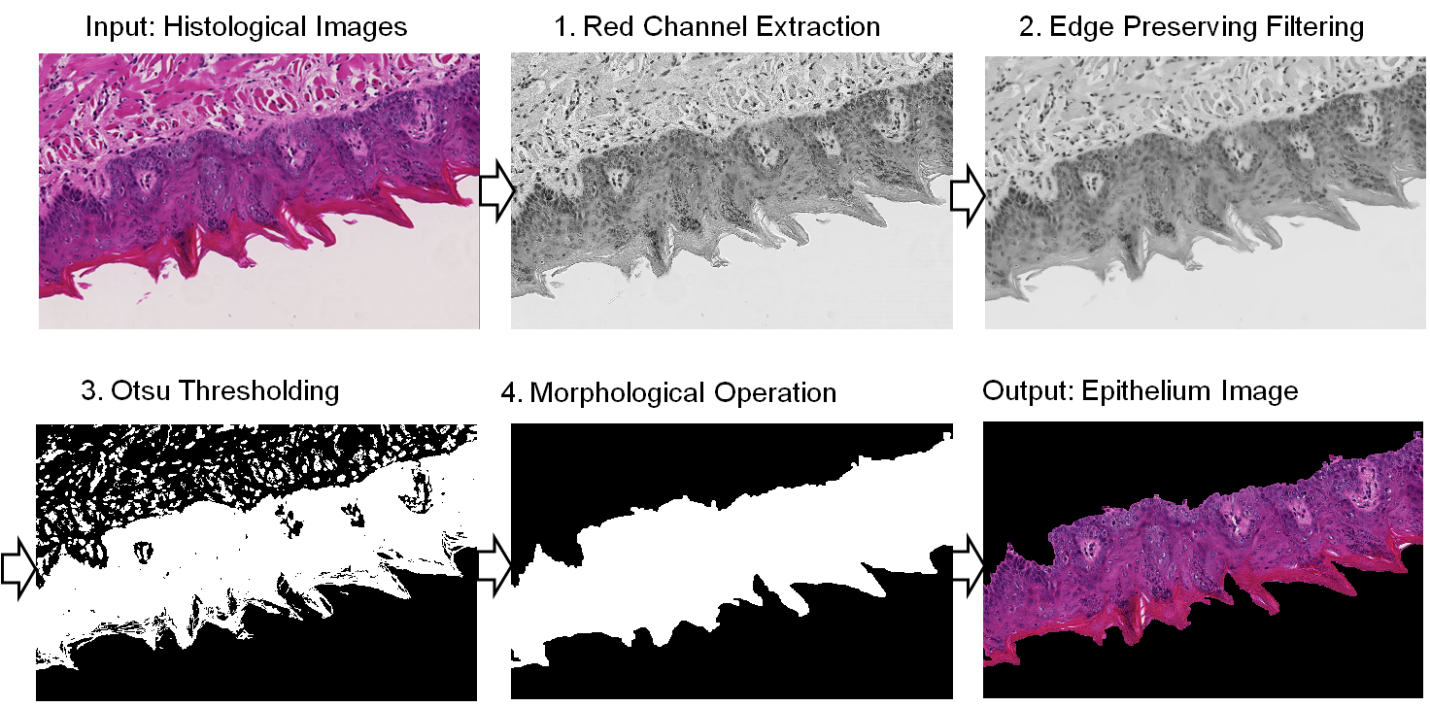


**Figure 1**. Segmentation of epithelium, nuclei, and cytoplasm


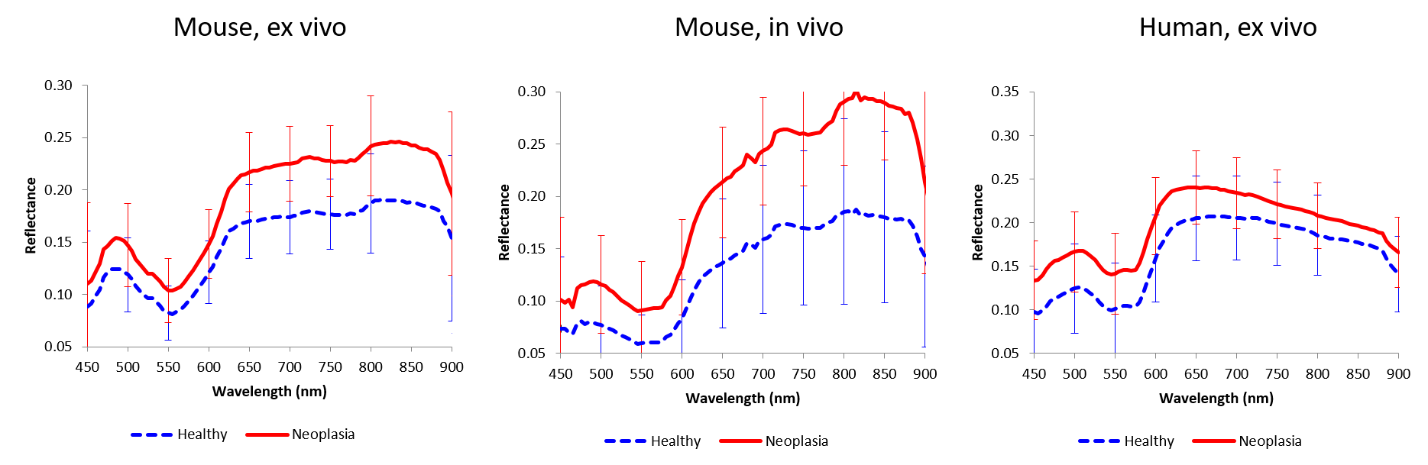


**Figure 2**. Comparison of ex vivo mouse, in vivo mouse and ex vivo human tissue spectra. Compared with ex vivo mouse spectra, the in vivo mouse spectra showed two dips at 542 nm and 577 nm owing to absorption from oxygenated hemoglobin (HbO2), suggesting more vasculature and more oxygenated hemoglobin in in vivo tissue than that in ex vivo tissue. Also there seems to be a larger spectral difference between normal and neoplastic tissue for in vivo tongue than for ex vivo tongue. The variations (standard deviation) of in vivo tongue is larger likely due to the tongue movement during imaging. The human spectra looks different from mouse spectra because human spectra was acquired from the side-view of specimen but not vertically from epithelium to deeper tissue depth as in mouse tongue. However, similar to mouse tongue spectra, the reflectance spectra of human tongue cancer showed higher reflectance intensity than normal tongue tissue
